# Shoulder Kinematic and Muscle Activity Compensations to Scapular Stabilizer Weakness: An Optimal Control Framework

**DOI:** 10.1007/s10439-025-03915-8

**Published:** 2026-03-04

**Authors:** Matthew S. Russell, Daanish M. Mulla, Peter J. Keir, Edward K. Chadwick, Dimitra Blana, Janessa D. M. Drake, Jaclyn N. Chopp-Hurley

**Affiliations:** 1 School of Kinesiology and Health Science, York University, 4700 Keele Street, Toronto, Ontario M3J 1P3 Canada; 2 Department of Kinesiology, McMaster University, Hamilton, Ontario Canada; 3 School of Engineering, University of Aberdeen, Aberdeen, Scotland UK; 4 School of Medicine, Medical Sciences and Nutrition, University of Aberdeen, Aberdeen, Scotland UK

**Keywords:** Muscle fatigue, Computer simulation, Upper limb, Kinematics

## Abstract

**Purpose:**

Shoulder kinematic and muscular redundancy promotes considerable variability, obscuring possible insights into neuromuscular control and compensation mechanisms for muscle weakness or fatigue. The current study harnessed recent advancements in optimal control formulations for computational musculoskeletal models to determine potential neuromuscular control strategies to compensate for isolated muscle weakness.

**Methods:**

A computational shoulder model characterized by independent clavicular, scapular, and humeral kinematics and 138 muscle elements was used. Optimal control-predicted thoracohumeral elevation kinematics were validated against published empirical kinematics. Force-generating capacity of the upper trapezius, middle trapezius, and lower trapezius, and serratus anterior were individually limited to 75%, 50%, and 25% maximal capacity to generate subsequent optimal control predictions of scapulothoracic kinematic changes associated with muscle weakness. Combined limited maximal force-generating capacity of lower trapezius and serratus anterior was also explored.

**Results:**

Model-predicted scapulothoracic kinematics showed good agreement with reference data, yet some significant differences were identified below 55° thoracohumeral elevation. Fatigue-mediated kinematic changes were most apparent during sagittal plane elevation. Serratus anterior weakness displayed the largest scapulothoracic kinematic changes at all thresholds of limited force-generating capacity. It also prompted the largest compensatory muscle activity changes from other shoulder muscles, while upper trapezius weakness prompted very little compensatory changes in muscle activity.

**Conclusion:**

Optimal control simulations were used to identify potential compensation mechanisms for shoulder muscle weakness and predict their effects on scapular kinematics. Findings suggest that thoracohumeral elevation in the scapular plane displayed less trapezius coactivity, both when ‘weakened’ and ‘unweakened.’ Thus, scapular plane tasks may isolate serratus anterior, while frontal plane tasks may achieve more balanced coactivation.

## Introduction

High interindividual (between individuals) and intraindividual (within an individual) variability in shoulder muscle activity and control precludes the ability to make inferences on the effects of targeted muscle stimuli on upper limb kinematics. This variability is likely consequent of the seemingly highly redundant muscular and kinematic degrees of freedom (DOF) at the shoulder that affords primates a wide landscape of potential solutions to nearly every upper limb task [1–4]. Scapulothoracic kinematic variability has challenged the characterization of normal/healthy and abnormal/unhealthy scapulothoracic kinematics and muscle synergies [5–8]. Recent works propose a critical perspective that muscle coactivation patterns associated with scapular stability (upper trapezius dominance, lower trapezius, and serratus anterior weakness) which were long considered to be ‘unhealthy’ by clinicians and researchers may in fact be a product of the morphological and anthropomorphic variability in scapular shape and muscle moment arms which promote a vast distribution of ‘normal/healthy’ shoulder muscle activity and kinematics [8–10].

A caveat of the inherent variability in scapulothoracic kinematics is that it can be difficult to characterize the kinematic contributions of individual shoulder muscles, which could otherwise help identify how the redundant set of shoulder joint effectors characterize the landscape of available motor control solutions. Muscle fatigue can sometimes provide an ecologically valid framework for assessing muscle control and compensation schemes in otherwise ‘healthy’ individuals, by observing how they respond to disruptions in muscle force-generating capacity [11–18]. This framework is complex and multifaceted, however, as several peripheral and central adaptations occur due to fatigue, which are difficult to fully characterize. Scapulothoracic kinematics changes associated with fatiguing stimuli – stimuli that would transiently reduce the force-generating capacity of muscles that attach to the scapula – are frequently reported in the literature [5, 17, 19–22]. However, interventions that attempt to fatigue an isolated set of one or more of the scapular stabilizer muscles (upper trapezius, middle trapezius, lower trapezius, and serratus anterior) [5, 23–27], rotator cuff muscles (supraspinatus, infraspinatus, subscapularis, teres minor) [17, 28], or tasks that stress the global shoulder musculature [22, 29–32] frequently report inconclusive findings, obscured by unclear trends swathed in variable responses. As we previously reported, individuals may even adopt opposing or bimodal scapulothoracic kinematic responses to fatigue following an overhead drilling task, illustrating how the landscape of redundancy in shoulder control may be so broad as to encompass completely opposing kinematic effects of fatigue [33]. While intraindividual variability is a less commonly reported metric, Mulla et al. [2018] provided further evidence of opposing kinematic changes with fatigue, reporting scapular plane kinematic differences within individuals of up to 10° in the opposite direction across successive completions of a rotator cuff fatigue task [17, 32, 33]. Similar levels of variability are consistent with other reports [22, 23, 34], which is considerable given that changes in static scapulothoracic angle of only 5° may be clinically meaningful for injury risk [35–37]. Importantly, such magnitudes of muscular and kinematic variability have the potential to obscure significant fatigue-related relationships in a very practical sense, by decreasing the calculated p-value of traditional hypothesis-based statistical tests.

While high variability may obscure shoulder muscle compensations to fatigue, comparisons of shoulder muscle fatigue-related effects across the literature are also complicated by the many different fatigue protocols employed. In addition, methodological limitations including the apparent error in skin-based scapular motion tracking [38, 39], EMG artifacts and errors between the sensor and the muscle of interest due to muscle movement underneath the skin [40], and the difficulty in capturing and characterizing the full breadth of muscles which contribute to shoulder motion due to their number, many partitions [41, 42], and depth [43] may limit a comprehensive understanding of the kinematic–muscular relationship at the shoulder.

Computational musculoskeletal modeling tools may offer comprehensive insights into the muscular-kinematic relationship at the shoulder. Such models replace observations of empirical data from an *in vivo* model with simulated data which are derived from a set of biomechanical equations, constraints, and assumptions about the biomechanical system [44, 45]. One of the most extensively cited computational shoulder models, the Delft Shoulder and Elbow Model (DSEM) [46], is well regarded for its anatomical fidelity, which has been bolstered with three decades of model validation and updates [47–50]. An implicit method for rapidly formulating optimal control problems as ordinary differential equations, harnessed with standard non-linear program solvers [51, 52], has been integrated with a current version of the DSEM to offer upper limb optimal control solutions within 6–24 hours [53]. Such timeframes were previously unachievable, hindered by mechanically stiff musculoskeletal elements which required timesteps 1500 times smaller to solve using explicit ordinary differential equations [50, 53]. Such advancements in optimal control formulation and stability make problems with low mass and high stiffness elements like the clavicle and scapula more accessible for exploring the muscle-kinematic relationship at the shoulder.

The purpose of this study was to use a computational musculoskeletal model of the shoulder to predict scapulothoracic kinematics changes associated with isolated reduced force-generating capacity of the scapular stabilizer muscles. A secondary aim of this study was to determine the model-predicted scapular stabilizer muscle activity compensations associated with isolated reductions in force-generating capacity of the scapular stabilizer muscles. We hypothesize that progressively reduced force-generating capacity will result in progressively altered scapulothoracic kinematics, and that scapular stabilizer muscle activity will progressively increase to compensate for simulated weakness.

## Materials and Methods

### Model Selection

A version of the DSEM which contains 138 muscle elements, characterizing 25 muscles of the shoulder and elbow, was used [47, 49, 50, 54]. The DSEM models thoracohumeral movements as motion across the sternoclavicular, acromioclavicular, and glenohumeral anatomical joints. The scapulothoracic gliding plane is modeled with elastic contact forces between the thorax ellipsoid and (1) the trigonum spinae and (2) the inferior angle. The contact force equation and its derivation are published elsewhere [55], but importantly this formulation generates very high forces if the contact points penetrate the thorax, yet are very low when the contact points egress beyond the perimeter of the thorax as to model soft tissue forces. Model degrees of freedom definitions are shown in Appendix A.

The DSEM is available open-source from the SimTK repository (https://simtk.org/projects/dsem) or from the Aberdeen Biomechanics and Biomedical Engineering GitHub Repository (https://github.com/AbdnBiomechEng) for use with OpenSim. The model-specific equations for musculoskeletal dynamics are previously described [53], therefore, the methodology will focus on the optimal control formulation and model validation.

### Degrees of Freedom and Optimal Control Solutions

In a multibody system where ***m*** actuators (muscles) exceed ***n*** kinematics DOFs, kinematic solutions are indeterminate [2]. Direct collocation methods can be harnessed to transcribe an optimal solution which satisfies muscular and kinematic constraints algebraically for a set of time-varying control trajectories (nodes) into a non-linear programming (NLP) problem. This formulation presents an optimal control problem that can be solved at all nodes simultaneously subject to optimizing a set of cost function criteria. Here, the control variable ***u*** represents the neural excitation signal to the muscle, which can be modeled as motoneuron activation/deactivation ***a*** through a non-linear function to describe the electro-chemical relationship [56]. Without these constraints on the control signals, control variables would not be physiologically feasible. The state vector ***x*** is dynamically consistent with the activation signal ***a***, regulated by the control signal ***u*** modeled within the system dynamic equation where ***x*** = (***q***, ***q̇***, ***s***, ***a***)^T^ defines the state vector. Here, ***q***, ***q̇*** represent generalized segment positions and velocities, respectively, and ***s*** represents the dynamic state (length) of the contractile muscle element governed by a force balance in the musculotendon complex in a three-element hill type model, defined previously at length [53]. Direct collocation methods discretize the continuous state (***q***) and control (***u***) trajectories of the system dynamics into *N* nodes of equal temporal spacing along the time duration of the motion, which become solvable with standard NLPs. Greater detail on the optimal control formulation is described at length in Appendix C and in previous research [53].

Adhering to kinematic and muscle activation constraints, we employed gradient-based NLP (IPOPT) subject to minimizing four terms: (1) mean-squared error of any input kinematic variables across nodes *∑(****qPred***_***i***_* – ****qMeas***_***i***_*)*^*2*^*N*, (2) the sum of the cube of the estimated muscle activations *∑(****a***_***i***_*)N *^*3*^ [57, 58], (3) a constraint term representing the mean deviation of the anterior face of the scapula from the wall of the thorax *∑(****Cscap****)N*, and (4) a constraint term representing the direction of the glenohumeral joint reaction force vector*∑(****Cgh****)N*. The cost function effectively seeks a solution that (1) minimizes the error between input and predicted kinematics, while minimizing (2) cubed muscle effort and penalizing (3) scapular kinematics that deviate from normative values, and (4) glenohumeral joint vectors that deviate from the glenoid and promote dislocation or impingement. The cubic weighting on muscle activity was previously found to promote distributed muscle activity across multiple muscles versus quadratic weighting that permitted fewer muscles with greater activation [59]. Constraints on scapular kinematics and glenohumeral stability were modeled as soft constraints, which are penalized for deviating in the cost function, rather than being modeled as hard constraints. Both the scapulothoracic constraint and the glenohumeral constraint have been described previously [50]. Each of these terms is subject to coefficients *w* that weight the relative error of each term at each node (N) in the discretized problem, such that the objective function appears as presented (Eq. 1):

1 $$\begin{array}{*{20}l} {\mathop {\min }\limits_{{\left\{ {{\boldsymbol{q}}_{i} ,\dot{\user2{q}}_{i} ,{\boldsymbol{u}}_{i} } \right\}_{i = 1}^{N} }} } \hfill \\ {\sum\nolimits_{i = 1}^{N} {\left[ {w1\cdot{\user2{q}}\user2{Pred}\left( i \right) - {\boldsymbol{qMeas}}\left( i \right)^{2} + w2 \cdot \sum\limits_{j = 1}^{m} {{\boldsymbol{a}}_{j} } \left( i \right)^{3} + w3 \cdot C_{Scap} \left( i \right) + w4 \cdot C_{GH} \left( i \right)} \right]} } \hfill \\ \end{array}$$

### Prescription and Validation of Predicted Model Solutions

Empirical scapulothoracic kinematic data acquired from transcortical pins into the scapula, tracked with electromagnetic motion sensors during full-range thoracohumeral elevation trials in the frontal plane, were provided by Ludewig and colleagues [60]. By comparison, model-predicted kinematics were produced by prescribing optimal control thoracohumeral elevation trials in the frontal, scapular, and sagittal planes of thoracohumeral elevation, which were defined as 0°, 40°, and 70° thoracohumeral plane of elevation angles, respectively. Sagittal plane model predictions were found to be increasingly unstable beyond 75° thoracohumeral plane of elevation. Angles approaching 90° in the thoracohumeral plane would result in simulations failing to converge after a maximum threshold of 10,000 iterations, or those that predicted scapulothoracic kinematics that were drastically different from empirical data. Thus, sagittal plane solutions were set with thoracohumeral plane of elevation angle of 70°.

Empirical elevation trials were prescribed from a resting posture with the arm hanging at the side to a maximal elevation that differed between participants. However, all participants achieved thoracohumeral elevation of at least 120° in each plane. Thus, model-predicted simulations were also prescribed from an initial resting position with the arm hanging at the side to a final thoracohumeral elevation of 120°. The prescription of model resting posture is detailed in Appendix B. Aside from the thoracohumeral angles set as input kinematic tracking variables, sternoclavicular elevation (SC_z) was tracked as an additional input variable using the average empirical sternoclavicular elevation data to constrain the high dimensionality of the available model solutions [60]. Model thoracohumeral elevation was explicitly prescribed by inputting an array of thoracohumeral and sternoclavicular joint angles at every 0.375 second interval to approximate a bell-shaped velocity profile [60, 61] over a 2.625 seconds-long motion. Thoracohumeral elevation to 120° was chosen as the target model thoracohumeral elevation as it was the minimum end range of motion exhibited by participants in the empirical dataset. While time-varying data were not available to infer thoracohumeral angular velocity, typical, goal-directed velocity profiles demonstrate a geometrically equivalent bell shape [61]. Thus, to normalize the velocity of the model-predicted elevations to the average velocity of the empirical dataset, thoracohumeral velocity began and terminated at 0°/s, peaking at 60°/s before ramping back to 0°/s at 2.625 seconds and 120° elevation. Direct collocation was set to achieve a solution in 16 nodes, spanning 2.625 seconds; thus, the interval between each node represented 0.175 seconds. As 16 nodes of resolution were set with only 8 input times, linear approximations were used to set initial guesses at nodes that did not coincide with a time point. The exact kinematic tracking terms that were input for each plane of thoracohumeral elevation are detailed in Table 1.

**Table 1:** Input kinematic tracking variables to prescribe model thoracohumeral elevation

Time (sec)	**TH_x**	**TH_y**	**TH_z**	Trunk_Hum_y			Trunk_Hum_z	Trunk_Hum_yy	SC_z			**EL_x**	**PS_y**
				Plane					Plane				
				*Fr*	*Sc*	*Sg*			*Fr*	*Sc*	*Sg*		
0	**0**	**0**	**0**	0	40	70	15	0	11	11	10	**0**	**5**
0.375	**0**	**0**	**0**	0	40	70	21.8	0	14	12	10	**0**	**5**
0.75	**0**	**0**	**0**	0	40	70	36.8	0	16	13	11	**0**	**5**
1.125	**0**	**0**	**0**	0	40	70	56.7	0	17	14	12	**0**	**5**
1.5	**0**	**0**	**0**	0	40	70	78.3	0	17	15	13	**0**	**5**
1.875	**0**	**0**	**0**	0	40	70	98.2	0	18	15	13	**0**	**5**
2.25	**0**	**0**	**0**	0	40	70	113.2	0	19	16	14	**0**	**5**
2.625	**0**	**0**	**0**	0	40	70	120	0	20	17	14	**0**	**5**

^*^ TH_x, TH_y, TH_z, EL_x, PS_y were locked

Where tracking variables differ by plane, columns *Fr*, *Sc*, and *Sg* indicate settings for frontal, scapular, and sagittal, respectively. Numbers provided are joint angles in degrees (°). Bolded, shaded columns indicate variables that were locked*, such that they cannot be altered in the optimal control solver.

### Validation of Model Predictions

A one-dimensional statistical parametric mapping (SPM) one-sample t test [62–64] was used to compare model-predicted scapulothoracic angles to a set of 12 empirically derived scapulothoracic joint angles [60] during dynamic thoracohumeral elevation trials in the frontal plane. For the following SPM models, optimal control-predicted scapulothoracic angle was the independent variable, and thoracohumeral elevation angle was the one-dimensional dependent continua. SPM analysis could only be conducted for thoracohumeral elevation in the frontal plane as this was the only plane of motion available for the empirically derived data.

We computed 12 frontal plane thoracohumeral elevation trials with all combinations of the following cost function (Eq. 1) weights: muscle effort (*w*_*2*_) weights of 1, 10, and 50, scapulothoracic constraint (*w*_*3*_) terms of 0.01, 0.1, and 1, and glenohumeral stability (*w*_*4*_) terms of 0.01 and 0.1. Kinematic tracking (*w*_*1*_), being the relative weight to which the optimal control solution can impose alterations in the input kinematic tracking variables (Table 1), was set to 100 for all cost function permutations. The terms *w*_*1*_ = 100 and *w*_*2*_ = 10 were previously found to produce reasonable predictions in an optimal controller cost function for gait prediction and thus guided the relative weights tested for our purposes of scapulothoracic control [53].

Empirical scapulothoracic joint angle data during thoracohumeral elevation was not available for the other planes of interest (scapular and sagittal plane), which limits our ability to perform a one-dimensional SPM analysis to determine predictive accuracy in these other planes. Therefore, the optimal control cost function weights that produced the best matching scapulothoracic model solution for thoracohumeral elevation in the frontal plane based on significant difference from the empirical dataset were then used to produce scapulothoracic kinematic predictions in the scapular and sagittal planes. Details on solver convergence and mesh density are shown in Appendix D.

### Reducing Force-Generating Capacity of Scapular Stabilizer Muscles

Following validation of the model performance in the frontal plane, and generation of model solutions in the scapular and sagittal planes, model solutions were regenerated in all three planes with maximal force-generating capacity (Fmax) of the scapular stabilizer muscles iteratively reduced to 75%, 50%, and 25%. Simulating muscle weakness in this way effectively increased the weighting of muscle activity on the ‘weakened’ muscle in the optimal control formulation (Eq. 1), while also setting a constraint on the maximal muscle force. As each muscle element in the model has a unique line of action and unique Fmax derived from cadaveric data, this method of simulating weakness was not proportional across all muscle elements. These model solutions were generated to simulate kinematic and muscular changes associated with upper trapezius weakness, middle trapezius weakness, lower trapezius weakness, serratus anterior weakness, and combined lower trapezius and serratus anterior weakness. Importantly, simulations of muscle weakness were prescribed by loading in the full solution of states and controls from the unweakened optimal control solution, rather than inputting kinematic states as described previously. This formulation for the problem permitted kinematics to deviate to find a new optimal control solution.

## Results

### Model Validation

**Fig. 1:**
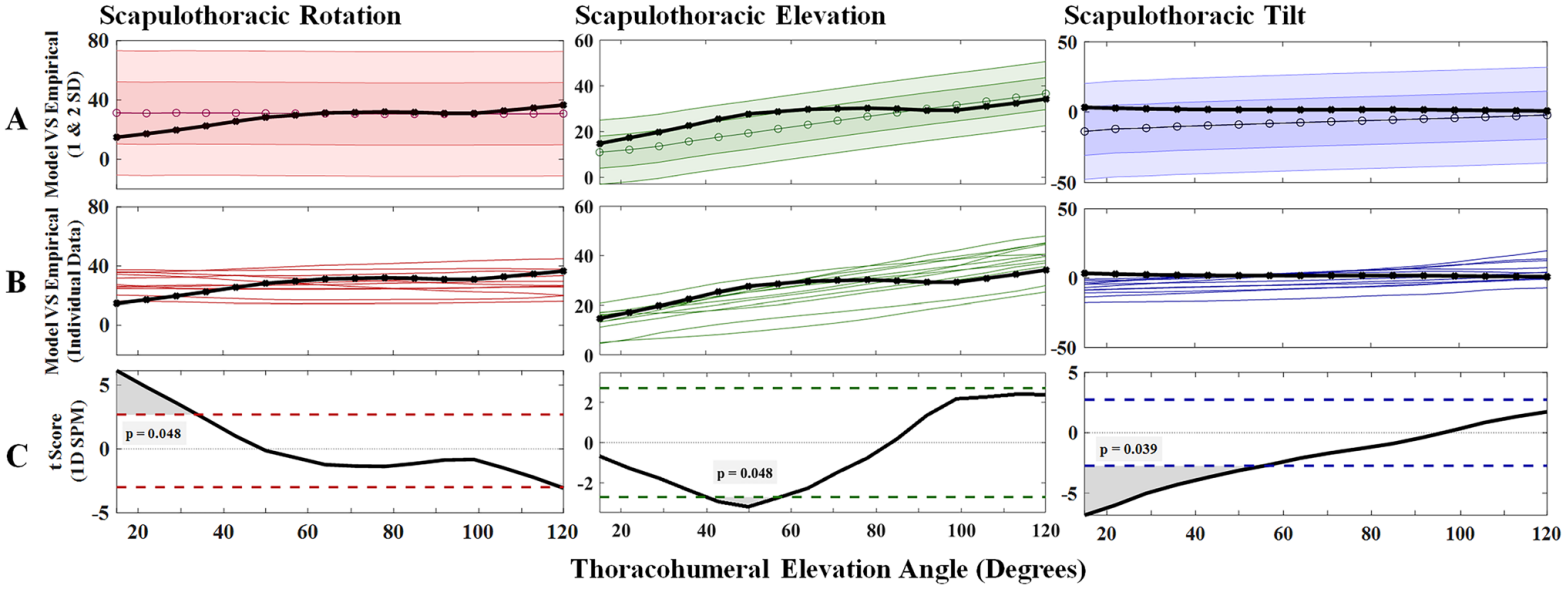
Model-predicted scapulothoracic joint angle optimal control solution for frontal plane thoracohumeral elevation from 15° to 120°. *Red*, *Green*, and *Blue* subplots represent scapulothoracic rotation, elevation, and tilt degrees of freedom, respectively. Subplots **A** represent model-predicted solution in black, bold, compared to empirical mean value in color. Shaded regions represent 1 standard deviation and 2 standard deviations. Subplots **B** represent model-predicted solution in black, bold, compared to the twelve individual participant scapulothoracic joint angles in color. Subplots **C** represent one-dimensional statistical parametric map t-statistic in black, bold, compared to t critical significance threshold in color, dotted

Testing of all permutations of cost function weights always achieved a solution with a region of significant difference along the continua of each scapulothoracic DOF. The solution with cost function weights *w*_*1*_ = 100, *w*_*2*_ = 10, *w*_*3*_ = 0.1, and *w*_*4*_ = 0.1 was the closest to achieving non-significant differences along the entire continua for all three scapulothoracic DOFs across all 12 cost function permutations tested. The resulting scapulothoracic kinematic solution is illustrated in Fig. 1 and compared to the mean and standard deviation of the empirical dataset [60]. The associated cost function values for all 3 planes of elevation are reported (Eq. 2):

2.1 $$\begin{aligned} Frontal Plane Cost Function:\,0.00988 & = 0.00011\left( {Kinematic Tracking} \right) + 0.00977\left( {Muscle Effort} \right) \\ & \quad + 0.00005 \left( {Scapular Constra{\mathrm{int}} } \right) + 0.0006 \left( {Glenohumeral Stability} \right) \\ \end{aligned}$$

2.2 $$\begin{aligned} Scapular Plane Cost Function:\,0.22122 & = 0.06502\left( {Kinematic Tracking} \right) + 0.16070\left( {Muscle Effort} \right) \\ & \quad + 0.00035 \left( {Scapular Constra{\mathrm{int}} } \right) + 0.00025 \left( {Glenohumeral Stability} \right) \\ \end{aligned}$$

2.3 $$\begin{aligned} Sagittal Plane Cost Function:\,0.38766 & = 0.18670\left( {Kinematic Tracking} \right) + 0.20097\left( {Muscle Effort} \right) \\ & \quad + 0.00011 \left( {Scapular Constraint} \right) + 0.00233 \left( {Glenohumeral Stability} \right) \\ \end{aligned}$$

### Limiting Maximal Muscle Force-Generating Capacity – Kinematic Changes

**Fig. 2:**
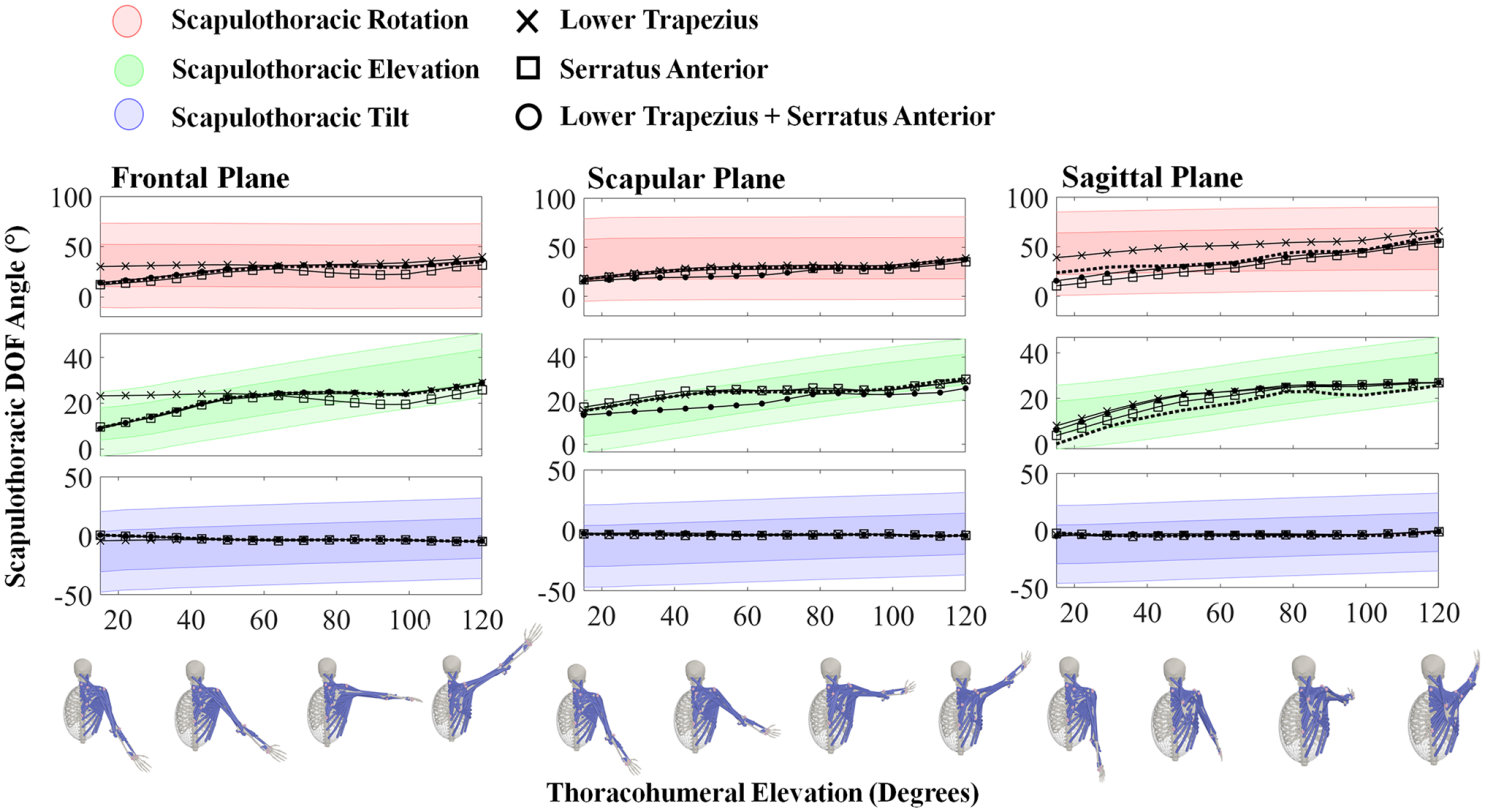
Model-predicted scapulothoracic joint angle optimal control solution for frontal plane, scapular plane, and sagittal plane thoracohumeral elevation from 15° to 120°. Solid black lines indicate the solution subject with Fmax of an isolated scapular stabilizer reduced to 25%, compared to the dotted line “no fatigue” solution. Simulation postures at 20°, 55°, 90°, and 120 thoracohumeral elevation

Model-predicted scapulothoracic kinematics changes were present when lower trapezius and serratus anterior muscle force-generating capacity was limited to 25% Fmax, yet 75% and 50% Fmax simulations did not always elicit an altered kinematic strategy, and upper and middle trapezius simulations did not reveal a unique kinematic strategy when limited to 25% Fmax. Thus, 25% Fmax simulations are illustrated for all three prescribed planes of motion for lower trapezius, serratus anterior, and combined lower trapezius–serratus anterior (Fig. 2), and 75% and 50% Fmax simulations are included in the supplementary material. Peak joint angle differences between 100% and 25% force-generating capacity simulations occurred between 0° and 60° of thoracohumeral elevation for lower trapezius and combined lower trapezius and serratus anterior weakness, while sole serratus anterior weakness simulations showed peak angular differentials primarily between 60° and 120° (Table 2).

**Table 2:** Peak joint angle differences between ‘unweakened’ and ‘25% max force-generating capacity’ simulations

Muscle	Frontal Plane			Scapular Plane			Sagittal Plane		
	*Rot*	*Elv*	*Tilt*	*Rot*	*Elv*	*Tilt*	*Rot*	*Elv*	*Tilt*
Upper Trapezius									
Middle Trapezius									
Lower Trapezius	15.5	12.9	–3.8				18.9	17.4	–1.4
Serratus Anterior	8.2	–4.5	–0.5	–4.0	1.5	–0.4	10.6	6.1	–0.7
Lower Trapezius + Serratus Anterior	–0.5	–1.2	–0.5	8.2	–5.9	1.5	–8.5	–7.4	–0.2

^*^Joint angles in degrees (°).

### Limiting Maximal Muscle Force-Generating Capacity – Scapular Stabilizer Muscle Activity Changes

**Fig. 3:**
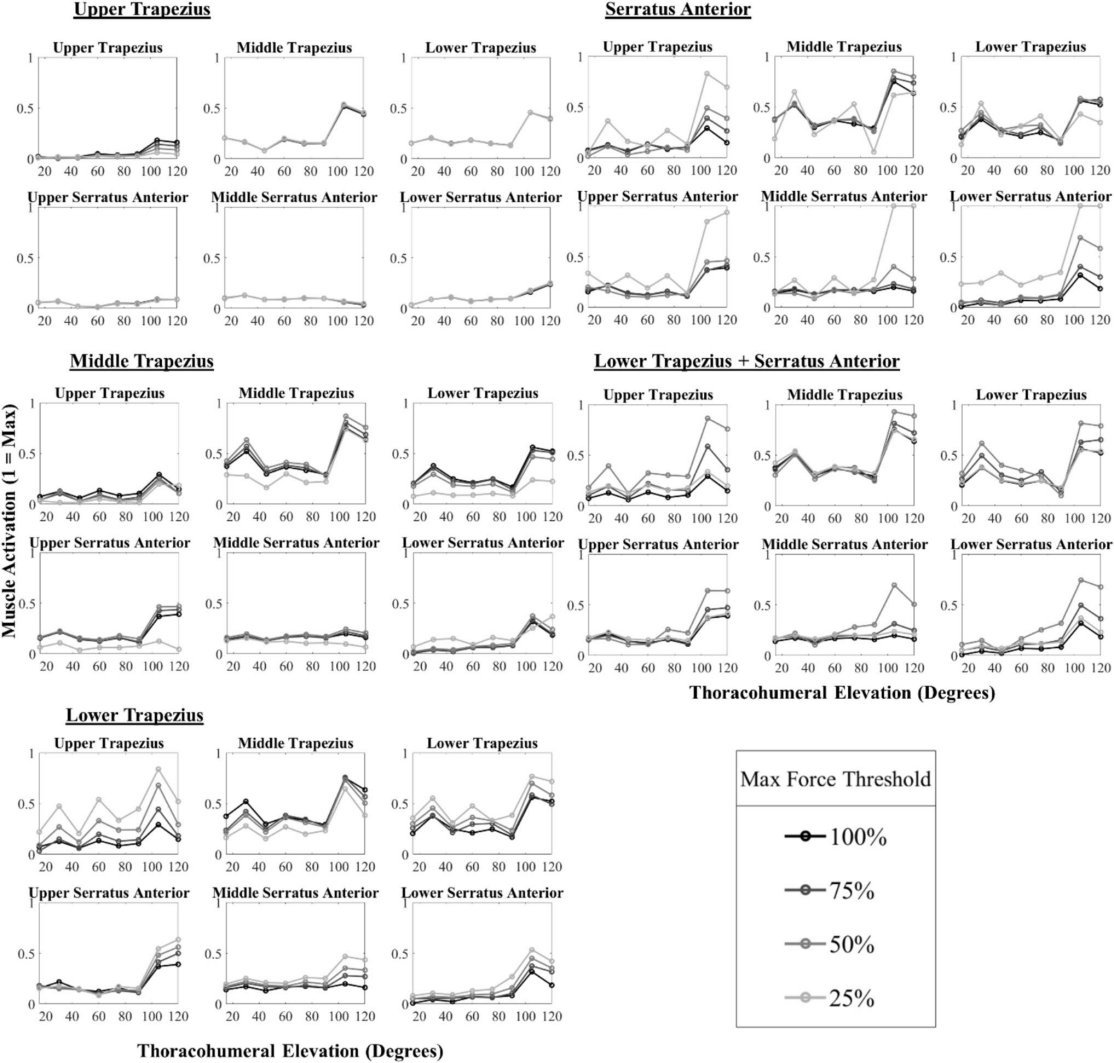
Predicted muscle activation during thoracohumeral elevation from 15° to 120° elevation in the frontal plane while limiting maximal force-generating capacity of scapular stabilizer muscles. Muscles with simulated weakness are bolded and underlined above respective simulation results

**Fig. 4:**
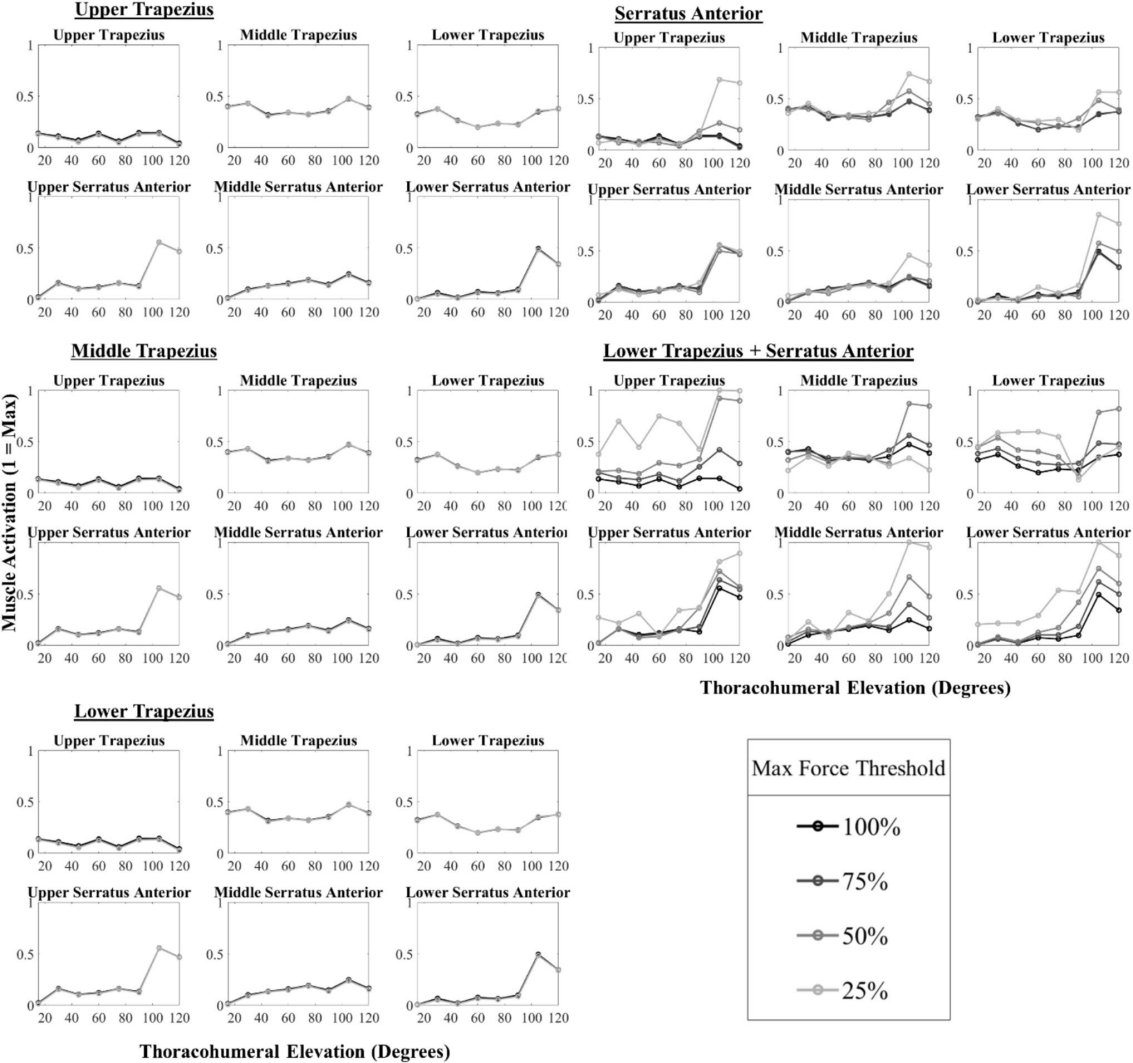
Predicted muscle activation during thoracohumeral elevation from 15° to 120° elevation in the scapular plane while limiting maximal force-generating capacity of scapular stabilizer muscles. Muscles with simulated weakness are bolded and underlined above respective simulation results

**Fig. 5:**
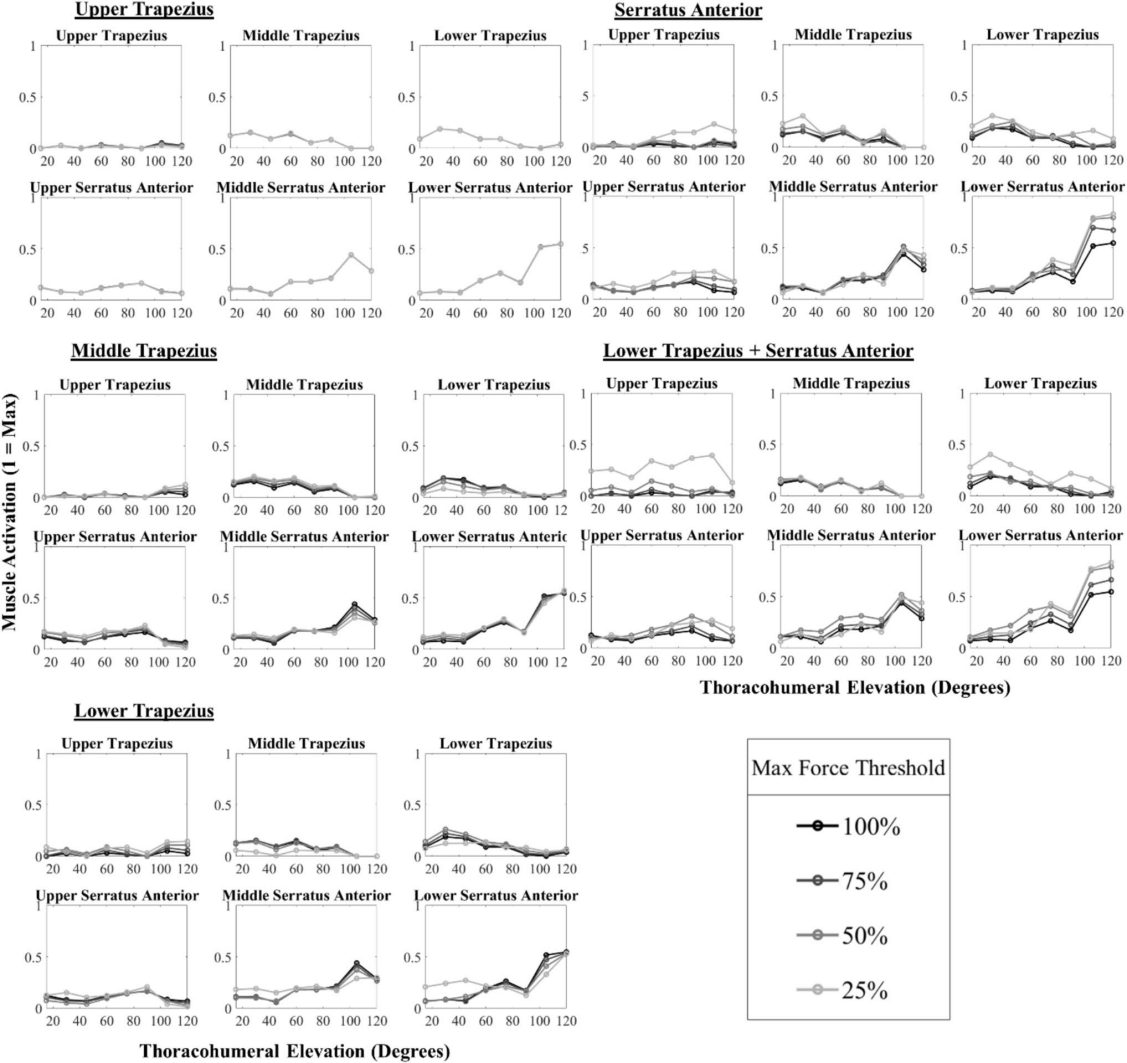
Predicted muscle activation during thoracohumeral elevation from 15° to 120° elevation in the sagittal plane while limiting maximal force-generating capacity of scapular stabilizer muscles. Muscles with simulated weakness are bolded and underlined above respective simulation results

Generally, scapular stabilizer muscle activity magnitude increased due to isolated muscle weakness. Increased fluctuation in muscle activity magnitude was also observed due to isolated muscle weakness; identifiable by increased prominence of peaks in the muscle activity predictions (Figs. 3, 4 and 5), where ‘muscle’ activations are the average activation of corresponding muscle elements (Appendix B). Scapular plane muscle activity changes appeared indifferent to trapezius weakness, with little change in activity in the sagittal plane and the most substantial muscle activity responses to trapezius weakness in the frontal plane. Similarly, weakness of serratus anterior prompted the greatest trapezius recruitment in the frontal plane. Upper trapezius weakness did not prompt muscle activity compensations (nor kinematics changes) in any plane. In almost all cases, peak activity for each muscle occurred at the same thoracohumeral elevation angle between 100% and 25% Fmax simulations. In some cases, certain peak muscle activations were reduced in 25% Fmax simulations compared to 100% (Tables 3, 4 and 5).

**Table 3:**
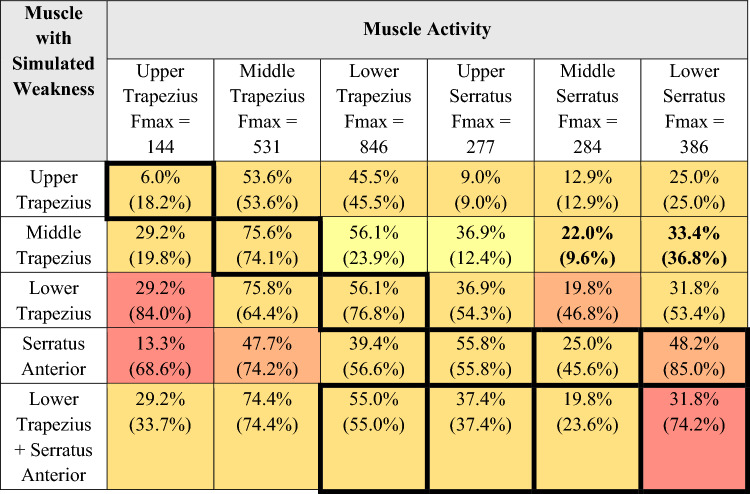
Peak muscle activity for each 100% force-generating capacity and 25% force-generating capacity simulation in the frontal plane. 100% capacity simulations on top, 25% capacity simulations in parenthesis below. Bolded numbers indicate a simulation where the 100% peak and 25% peak did not occur at the same thoracohumeral elevation angle. Fmax in Newtons, boxes indicating activity of muscles with reduced Fmax are outlined

**Table 4:**
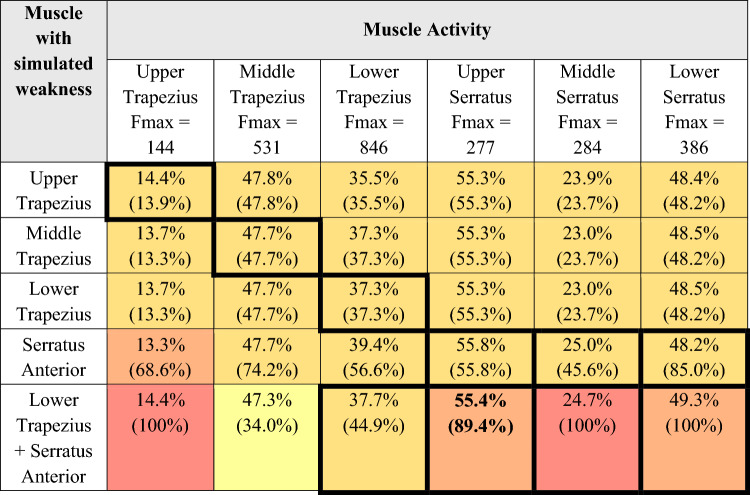
Peak muscle activity for each 100% force-generating capacity and 25% force-generating capacity simulation in the scapular plane. 100% capacity simulations on top, 25% capacity simulations in parenthesis. Bolded numbers indicate a simulation where the 100% peak and 25% peak did not occur at the same thoracohumeral elevation angle. Fmax in Newtons, boxes indicating activity of muscles with reduced Fmax are outlined

**Table 5:**
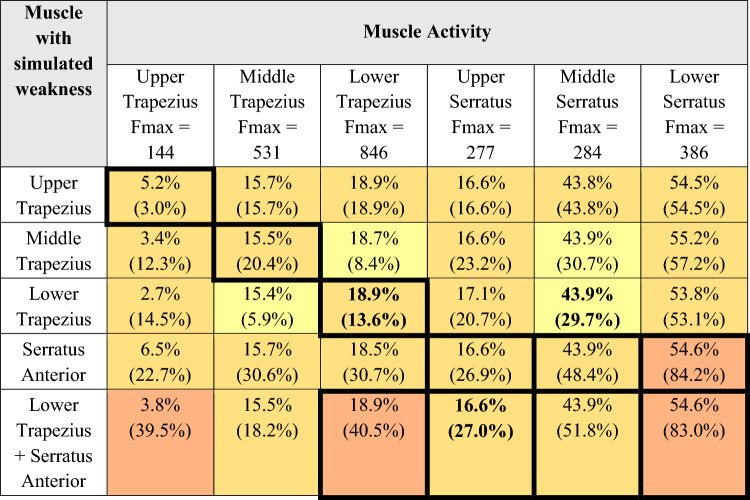
Peak muscle activity for each 100% force-generating capacity and 25% force-generating capacity simulation in the sagittal plane. 100% capacity simulations on top, 25% capacity simulations in parenthesis. Bolded numbers indicate a simulation where the 100% peak and 25% peak did not occur at the same thoracohumeral elevation angle. Fmax in Newtons, boxes indicating activity of muscles with reduced Fmax are outlined

## Discussion

This study used a computational shoulder model to predict scapulothoracic kinematic changes associated with reductions in force-generating capacity of isolated scapular stabilizer muscles. When model-predicted kinematics were validated against empirical kinematics from frontal plane thoracohumeral elevation, one region of significant difference was identified along the predicted kinematic continua of each scapulothoracic DOF; each below 55° of TH elevation. Kinematic predictions otherwise did not exceed the threshold to achieve a significant difference compared to the empirically derived scapulothoracic angles. When modeling scapulothoracic kinematics changes associated with muscle weakness, predicted kinematic solutions showed changes at 25% Fmax of most scapular stabilizers when compared to the unweakened (100% Fmax) kinematics. Muscle activity typically increased across all scapular stabilizer muscles as muscle force-generating capacity was progressively weakened. A more balanced muscle recruitment strategy in the frontal plane and a more serratus anterior isolating strategy in the scapular plane also emerged with reduced Fmax. Some scapular plane kinematic and muscle solutions even appeared wholly unaffected by reduced upper and middle trapezius Fmax. Summarily, we reject our hypothesis that kinematics changes would become progressively more apparent with simulated weakness when any scapular stabilizer had reduced Fmax, but we generally accept our hypothesis that muscle activity would progressively increase to compensate.

### Model Validation

SPM analysis of model-predicted kinematics compared to criterion empirical kinematics revealed regions of statistically significant kinematics differences between 15° and 30° for scapulothoracic rotation, between 40 and 55° for scapulothoracic elevation, and between 15° and 55° for scapulothoracic tilt. These significant regions had p-values close to the alpha threshold of 0.05, however, with values of p = 0.048, p = 0.048, and p = 0.039, respectively. The small region of significance observed for scapulothoracic elevation (Fig. 1) may suggest that scapulothoracic elevation is more sensitive than scapulothoracic rotation and tilt for achieving optimal thoracohumeral elevation mechanics based on the current cost function; thus, an optimal model solution could conceivably be achieved while permitting significant rotation and tilt differences compared to the empirical dataset.

### Limiting Maximal Muscle Force-Generating Capacity – Kinematic Changes

Overall, serratus anterior was the only muscle to alter kinematics across all three planes of motion when weakened. These kinematic changes typically demonstrated increased scapulothoracic external rotation ranging from − 4.0° to − 10.6° depending on the plane. However, changes in scapulothoracic elevation were also apparent, particularly in the frontal (− 4.5°) and sagittal (6.1°) planes. Scapulothoracic tilt did not change in most muscle weakness simulations, with the largest peak angular differential being − 3.8° due to lower trapezius weakness in the frontal plane. Increased scapulothoracic external rotation associated with weakness of serratus anterior may be an important consideration for increased subacromial space width, a factor which reduces subacromial impingement risk [24, 65, 66]. Conversely, frontal and sagittal plane kinematics solutions with 25% Fmax-limited lower trapezius displayed increases in scapulothoracic internal rotation, particularly below 60° thoracohumeral elevation, which would act to reduce subacromial space width and increase impingement risk within the critical range of thoracohumeral elevation that is associated with reduced subacromial width and pain [24, 65, 66]. However, these kinematic changes associated with weakened lower trapezius were paired with observed increases in scapulothoracic elevation, which are considered protective against subacromial impingement and rotator cuff tears [24, 65, 66]. Thus, while lower trapezius weakness appeared to promote scapulothoracic kinematic changes, it is difficult to ascertain whether the net effect of these kinematic changes is protective or harmful.

In simulations where lower trapezius and serratus anterior were both weakened, kinematic changes resembled those that appeared when only serratus anterior was weakened, particularly with respect to scapulothoracic rotation. This could suggest that weakness of serratus anterior may have the greatest effect on scapulothoracic motion of any single scapular stabilizer muscle during scapular motions in the frontal and scapular planes. The unique capacity of the serratus anterior has been highlighted for its ability to contribute to all three scapulothoracic DOF during thoracohumeral elevation by generating upward rotation, posterior tilting, and external rotation [67, 68]. Previous work leveraging a similar mechanistic modeling framework with unique inverse dynamic simulations of subsets of muscles to simulate functionally electrically stimulated muscles reported that serratus anterior on its own can maintain most critical scapulothoracic movements [69]. The underlying shoulder model used in this previous study is the same as the current; thus, the current model architecture suggests serratus anterior may be able to drive many scapulothoracic functions necessary for activities of daily living from a purely mechanistic perspective. However, these phenomena are unlikely to be valid ecologically, as muscle coactivation strategies are a well-known method in motor control for achieving stability [33, 70–72]. Yet, serratus anterior has the largest moment arm of any scapular stabilizer muscle, thereby making it mechanically advantageous for recruitment among the redundant shoulder muscle DOF [67, 68]. Chiefly, reductions in serratus anterior muscle activity are associated with abnormal and subclinical scapular motion, reported to often produce a ‘shrugged’ or elevated and externally rotated scapula that can present with pain [21, 65, 73, 74]. This analysis offers evidence that insufficiency in serratus anterior force production during dynamic motion appears to enable some of the largest changes in model-predicted kinematics. The potential contribution of scapulothoracic contact forces against the wall of the thorax and the surrounding soft tissues is modeled in our simulations and optimized within the cost function. These forces were found to be less than 1N in all of our simulations and are reported in Appendix E.

### Limiting Maximal Muscle Force-Generating Capacity – Scapular Stabilizer Muscle Activity Changes

Model-predicted scapular stabilizer muscle activity typically increased when any selected scapular stabilizer muscle was weakened, with two exceptions. First, the upper trapezius had no effect on kinematic changes, and little to no muscle activity changes in the scapular and sagittal planes with no weakness-mediated muscle activity increases greater than 2.5%. Second, no kinematics changes were observed when trapezius partitions were weakened during scapular plane elevation, and muscle activity changes during these simulations did not differ by more than 0.7% compared to an unreduced Fmax (Table 2). It is challenging to compare these two phenomena in our results against empirical evidence from the literature since many ecological pathways for muscle weakness come with limitations. Fatigue stimuli are unlikely to truly isolate the desired muscle in such a redundant system [17, 21, 23, 34] while muscle imbalances due to clinical or athletic adaptations are not expected to have the same control policies as healthy individuals [2, 25, 26]. These are some of the limitations which in fact compelled this study. Inferences from EMG [75], cadaveric research [76], and muscle modeling [77], however, concur on the actions of the trapezius, where upper partitions connect to the clavicle and perform elevation, middle partitions primarily retract, and lower partitions are thought to be the only region that can significantly contribute to external rotation for arm elevation [7]. Thus, from a purely mechanistic perspective, these muscle actions may explain why our model found upper and middle trapezius Fmax reductions to be minimally impactful for arm elevation. Other shoulder tasks, however, would likely reveal important mechanistic changes associated with reduced Fmax of these muscles.

Across the three planes of thoracohumeral elevation, frontal plane elevation typically demonstrated the greatest overall peak activity across the trapezius and scapular stabilizers ranging from 25.3% to 42.2% peak activity, which appeared reduced in the scapular plane and further reduced in the sagittal plane, which ranged from 25.3% to 25.8% peak activity. These findings could be important for consolidating our understanding of scapular stabilizer muscle activity and fatigue. For instance, in the context of studies which seek to stimulate comprehensive scapular stabilizer muscle fatigue, the current study suggests that frontal plane thoracohumeral elevation tasks may be best suited for a balanced contribution of all muscles. These findings also help make sense of current trends in literature which have found some success in fatiguing the scapular stabilizers in the frontal plane [5, 22, 78], yet fatigue stimuli in other planes appear less successful or preferentially fatigue serratus anterior [17, 23, 27, 30].

### Limitations

As a primarily mechanistic model of shoulder movement and weakness (reduced force-generating capacity), the findings of this study are subject to several assumptions which should be considered. Firstly, optimal weighting of terms in the cost function was tested and validated purely for frontal plane thoracohumeral elevation in a non-weakened state. The optimization function serves as an analog for the central nervous system’s strategy to minimize several possible criteria [79–85]. The present study assumes that optimization criteria remain relatively stable in the presence of muscle weakness, yet future research may improve our understanding of fatigue-related kinematic and muscular compensation by investigating whether optimization criteria change *in vivo* due to muscle weakness or fatigue. In particular, the transient state of fatigue is highly sensitive to the time history of muscle activity, and as other models [86] have been validated in predicting the complex motor unit behaviors during muscle fatigue, future research may be able to formulate more ecologically valid fatigue simulations that can pose meaningful reflections of human optimization criteria using fatiguing tasks. These same considerations hold true for any population with altered function, motor control, or sensation as the current research determined cost function weights inductively from validations against unaffected healthy individuals [60]. On the topic of simulating muscle ‘weakness’ or ‘fatigue,’ it is important to again reflect on the mechanistic nature of the model, which does not characterize the sensory contributions to motor control that surely contribute to an ecologically valid system. Due to challenges regarding trapezius and serratus anterior EMG collection during dynamic arm raising tasks which are sensitive to muscle kinematics changes, the current study opted to infer model validity through comparisons with empirical kinematic data and assume that kinematic validity reflects reasonable muscle activity estimates [31–33]. Model instability near 90° thoracohumeral plane of elevation necessitated that 70° be set as the simulation analog for the ‘sagittal plane,’ which may have arisen due to muscle-element wrapping issues and subsequent discontinuities in moment arm lengths. Readers are recommended to not apply the results of the ‘flexion’ or ‘sagittal plane’ simulations as a truly valid representation of in vivo muscle control in a true sagittal plane arm elevation, if for no other reason than the limitations described here. These results, however, do provide contrasts on plane of humeral elevation angle effects on shoulder muscle control. Similarly, problem convergence became less stable for muscle weakness simulations at 16 nodes. The least stable simulations were when LT, SA, or both muscles’ max force was limited to 25%. Thus, reducing the nodes for weakness simulations made convergence achievable within 24–72 hours for weakness; a 3–4x increase compared to unweakened simulations which took approximately 6–12 hours. The statistical implications of an empirical sample size of N = 12 should be considered, as this dataset likely lacks a complete characterization of the full landscape of valid empirical scapulothoracic kinematics. The DSEM itself represents the musculoskeletal features derived from a single human cadaver [46]; thus, any accurately predicted values are likely to fall somewhere within the landscape of feasible kinematic solutions but are unlikely to approximate the empirical mean.

### Optimal Control Simulations

Overall, lower trapezius and serratus anterior emerged as two key muscles that may support several functional arm movements, but with key differences. Serratus anterior, specifically the lower fibers, are a mechanically advantageous upward rotator of the scapula [7, 77]. However in our simulations, weakness of serratus anterior often permitted excess internal rotation of the scapula. This muscle was also highly active in overhead postures and in scapular plane motions. Lower trapezius did not appear as critical to the scapular plane or overhead arm postures; yet, weakness of this muscle was associated with large kinematic changes in the frontal and sagittal planes, particularly below 90° arm elevation. The magnitude of these kinematic changes in Fig. 2 may reflect the large peak (Fmax) force-producing capacity of this muscle compared to the other scapular stabilizers. The functional capacity of both of these two scapular stabilizers may therefore be tantamount to maintaining functional upper limb capacity, and weakness through injury, surgery, or other factors may result in significant impairments. These findings bolster previous research on the importance of these muscles for shoulder rehabilitation [26, 68, 73, 74, 87].

While optimal control simulations have shown previous success in modeling postural maintenance and gait, this study is one of the first to predict accurate scapulothoracic kinematics. Central to this feat is the operationalization of a cost function that could accurately predict scapulohumeral rhythm, which required systematic tuning of cost function analogs for scapulothoracic kinematics and glenohumeral stability. Anecdotally, cost function tuning revealed that the relative balance of muscle effort, scapulothoracic adherence, and glenohumeral stability were sensitive to achieving realistic solutions. A disproportionately high weighting of muscle effort often minimized scapulothoracic motion while consequently producing elevated arm positions that would dislocate the humeral head to achieve elevation, while high scapulothoracic adherence weights would produce excessive tilt and rotation during elevation, and high GH stability weights often promoted excess scapulothoracic elevation to provide the humerus with a more stable support. While we do not know the actual ‘weight’ of these factors during normal and ‘weakened’ arm trajectory planning, accurate model outputs themselves may help to determine accurate modeling of the many determinants in CNS motor planning. Therefore, the utility of optimal control predictions of human motion may be twofold, where they can (1) be used to proactively simulate the full dynamics of human movement; useful for probing questions that cannot be assessed *in vivo,* and (2) may subsequently help confirm which variables support motor planning.

### Conclusion

This study presents a model and optimal control framework for producing scapulothoracic kinematic solutions that closely match gold-standard empirical data. The notion of isolating a single scapular stabilizer with reduced force-generating capacity is not physiologically possible; however, reducing the maximal force-generating capacity of scapular stabilizers with this modeling framework provided several insights on shoulder muscle compensation strategies, which may reveal insights on muscle weakness and fatigue. Serratus anterior weakness seems to demonstrate particular importance in maintaining normal scapular kinematics. Results indicated that serratus anterior muscle recruitment may be isolated best in the scapular plane (40° anterior to the frontal plane) compared to the sagittal and frontal planes. While upper trapezius and lower trapezius weakness prompted little change in compensatory shoulder muscle activity, lower trapezius and serratus anterior appeared to reach their highest activity above 100° of thoracohumeral elevation, which may contribute to the risks of shoulder muscle fatigue and kinematic changes associated with overhead work. The validity of model-predicted kinematics and muscle activity changes to weakness and fatigue would benefit from a deeper understanding of the somatosensory effects and other *in vivo* changes to the central nervous system’s optimization criteria.

## Figures and Tables

**Fig. 6:**
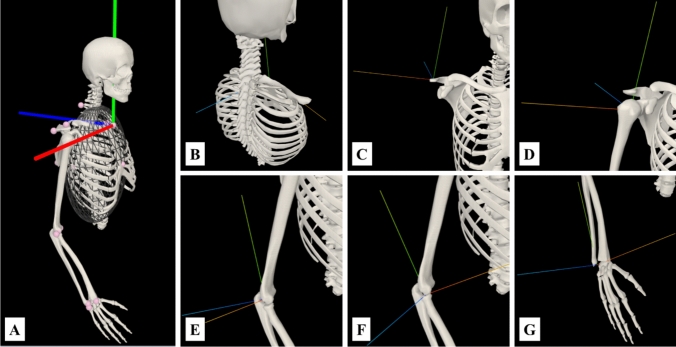
Visualization of the model global coordinate system and the local coordinate system of each rigid body in the model. X-axis is illustrated in red, Y-axis is illustrated in green, and Z-axis is illustrated in blue. **A**. The global coordinate system superimposed over the thorax local coordinate system – they are identical in this frame **B**. The local coordinate systems of the clavicle C. scapula D. humerus E. ulna F. radius G. hand

**Fig. 7:**
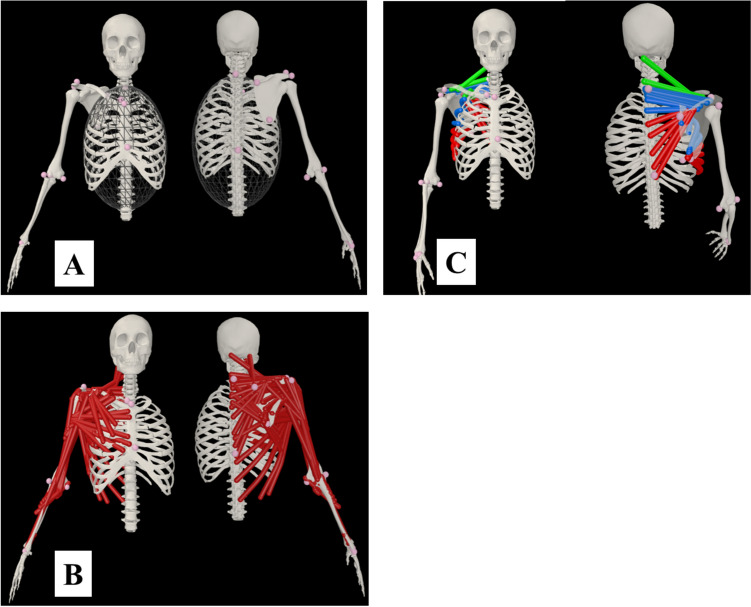
Visualization of statically optimized “hanging-arm” posture in OpenSim without (**a**) and with (**b**) visualization of all muscle elements. Used as initial guess for direct collocation. **c** Scapular and clavicular model geometry set to 75% opacity; muscle elements of trapezius and serratus anterior color-coded by partition: upper (green), middle (blue), lower (red)

**Fig. 8:**
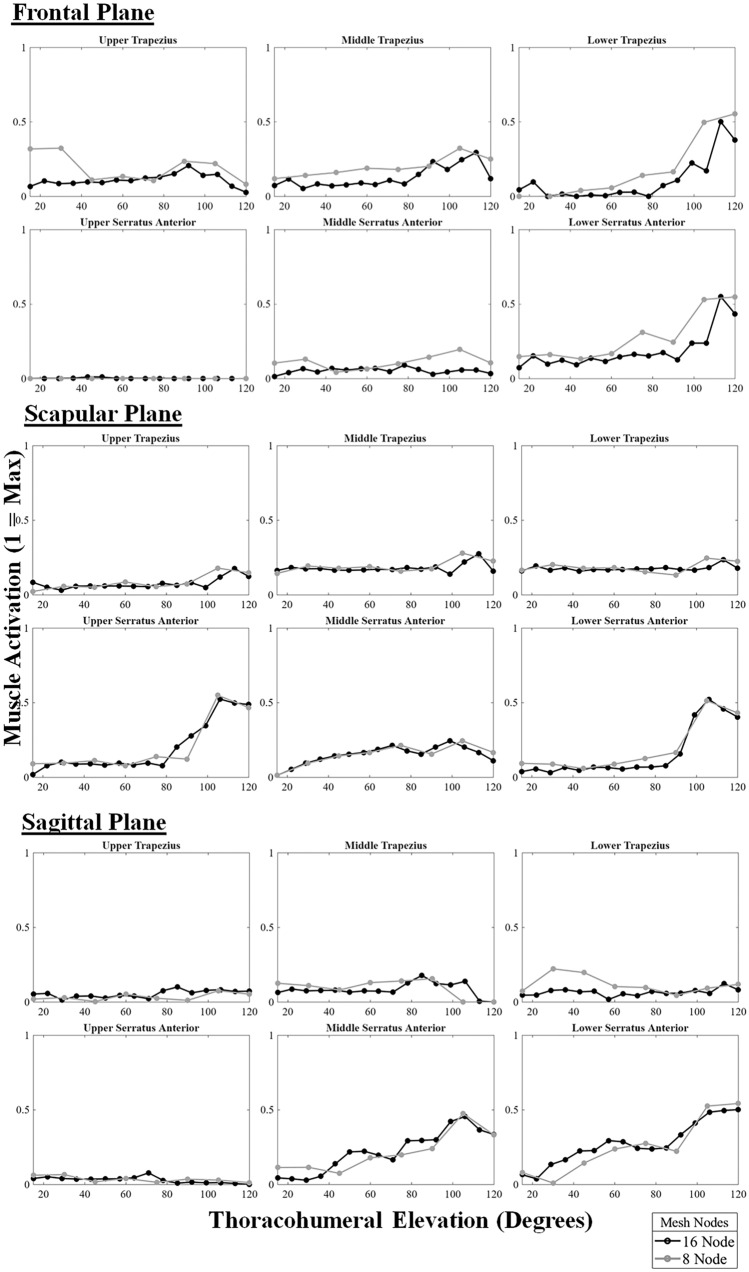
Scapular stabilizer muscle activation predictions between eight and sixteen node mesh resolution

**Fig. 9:**
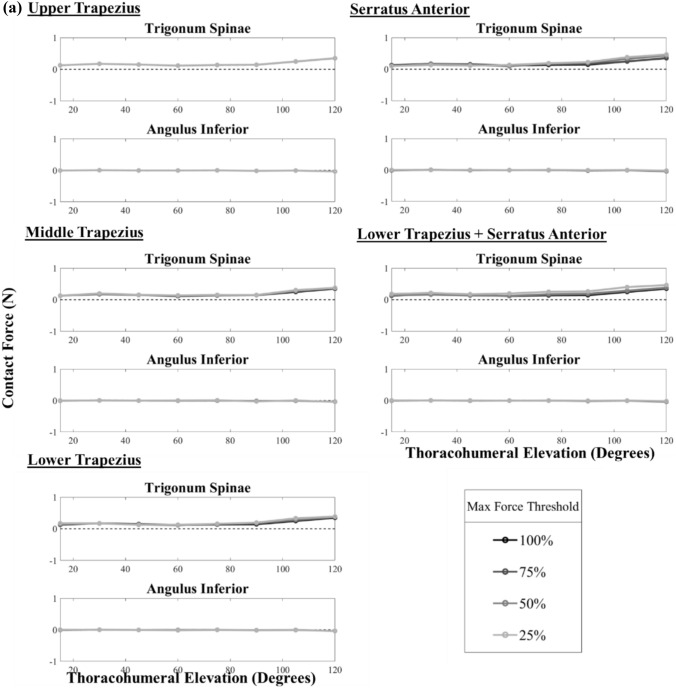

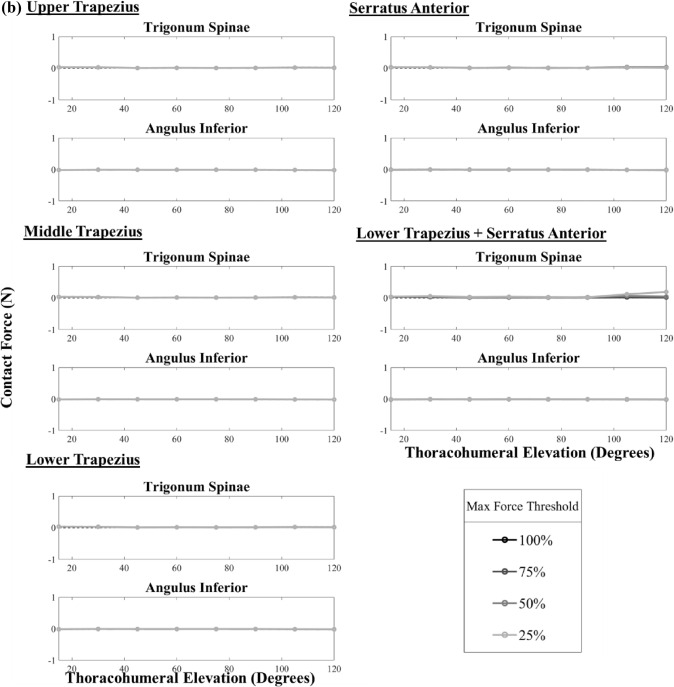

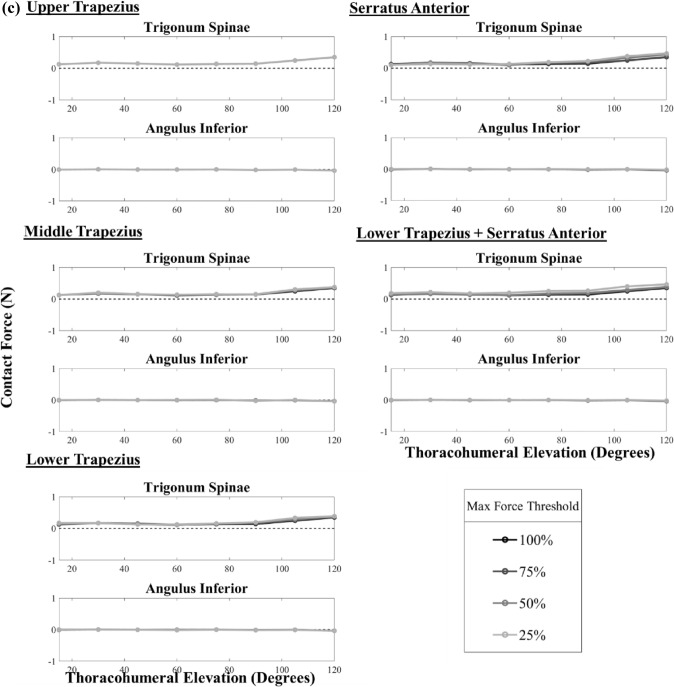
**A**: Scapulothoracic contact forces during thoracohumeral elevation to 120° in the frontal plane. **B**: Scapulothoracic contact forces during thoracohumeral elevation to 120° in the scapular plane. **C**: Scapulothoracic contact forces during thoracohumeral elevation to 120° in the sagittal plane

## Data Availability

Data are available upon reasonable request.

## Model Coordinate Definitions

Provided below are the joint angle coordinates as defined in the DSEM, and how they align with ISB recommended definitions (Table 6). Importantly, LCS definitions only change nominally across both methods, and orientations are consistent (Fig. 6). However, the DSEM does not expressly model scapulothoracic joint angles; thus, these angles were calculated using ISB recommended definitions (Table 7).

**Table 6:** Model degrees of freedom definitions and equivalent degree of freedom (DOF) notation as recommended by the International Society of Biomechanics [88]

Model anatomical DOF	Equivalent ISB DOF	ISB DOF definition
TH_x	*e*1*:* α_GT_	Thorax flexion about the Z-axis of the global coordinate system
TH_y	*e*3: γ_GT_	Thorax axial rotation about the Y-axis of the global coordinate system
TH_z	*e*2*:* β_GT_	Thorax lateral flexion about the X-axis of the global coordinate system
SC_x	*e*3: α_SC_	Sternoclavicular axial rotation about the Z-axis of the clavicular local coordinate system
SC_y	*e*1*:* γ_SC_	Sternoclavicular protraction about the Y-axis of the proximal thorax local coordinate system
SC_z	*e*2*:* β_SC_	Sternoclavicular elevation about the common axis perpendicular to *e*1 and *e*3 of the sternoclavicular joint
AC_x	*e*3: α_AC_	Acromioclavicular tilt about the Z-axis of the scapular local coordinate system
AC_y	*e*1: γ_AC_	Acromioclavicular retraction about the Y-axis of the proximal clavicle
AC_z	*e*2: β_AC_	Acromioclavicular medial rotation about the common axis perpendicular to *e*1 and *e*3 of the sternoclavicular joint
GH_y	*e*1: γ_GH1_	Glenohumeral plane of elevation (POE) about the Y-axis of the scapular local coordinate system
GH_z	*e*2: β_GH_	Glenohumeral elevation about the X-axis of the humerus
GH_yy	*e*3: γ_GH2_	Glenohumeral internal or axial rotation about the Y-Axis of the humerus
Trunk_Hum_y	*e*1: γ_h_	Thoracohumeral plane of elevation (POE) about the Y-axis of the thoracal local coordinate system
Trunk_Hum_z	*e2*: β_h_	Thoracohumeral elevation about the X-axis of the humerus
Trunk_Hum_yy	*e3*: (γ_h_)_2_	Thoracohumeral internal rotation about the Y-axis of the humerus
EL_x	*e*1: α_HF0_	Elbow flexion about the Z-Axis of the proximal humeral local coordinate system
PS_y	*e*3: γ_HF_	Elbow pronation or axial rotation of the forearm about the Y-axis of the forearm local coordinate system

**Table 7:** Scapulothoracic degrees of freedom defined using International Society of Biomechanics recommended notation [88]

ISB DOF	ISB DOF Definition
*e*1: γ_S_	Scapular retraction about the Y-axis of the thorax local coordinate system
*e2*: β_S_	Lateral or medial rotation of the scapula about the axis defined by *e*_1_ × *e*_3_
*e3*: α_S_	Anterior or posterior tilt about the Z-axis of the scapular local coordinate system

## Statically Optimized Initial Posture

To ensure that optimal control predictions initiated from reasonable estimates, the initial resting position of the arm was determined from a statically optimized posture that was set to minimize muscle activity. This posture was defined by providing a range of upper and lower bound joint angle limits to ensure that the static optimization function searched for a posture that minimized muscle stress while maintaining that the arm would be hanging at the side of the body. The final solution for the statically optimized hanging-arm posture is provided (Table 8; Fig. 7). Importantly, in the operationalization of direct collocation, the initial arm state described here did not necessarily match the first frame (hanging-arm) of the solution motion in any plane of elevation. Rather, the initial arm state described acted as an initial guess for all three ‘unweakened’ solutions, ensuring that the initial guess was dynamically feasible.

**Table 8:** List of statically optimized model joint angles to minimize muscle effort during a static arm hang

Model Degree of Freedom (DOF)	Statically Optimized Joint Angle	Lower and Upper bounds of Static Optimization Joint Angle Limits
TH_x	0°	
TH_y	0°	
TH_z	0°	
SC_x	11.3°	6° ; 12°
SC_y	–20.4°	–24° ; –19°
SC_z	21.3°	10° ; 30°
AC_x	–6.2°	–10° ; 5°
AC_y	39.9°	33° ; 43°
AC_z	–10.1°	–15° ; 0°
GH_y	–10.0°	–10° ; 10°
GH_z	0.7°	–10° ; 10°
GH_yy	–9.9°	–10° ; 10°
EL_x	0.9°	0° ; 20°
PS_y	73.1°	70° ; 90°

Upper trapezius partition was selected as the model muscle elements inserting on the clavicle, whereas elements attaching to the scapula were defined as middle and lower partitions [54, 76, 89] (Fig. 7). Of the 11 ‘scapular’ trapezius elements, the 6 most superior elements were classified as representative of middle trapezius for the current simulations, and the bottom 5 were classified as representative of lower trapezius (Fig. 7), following best practices for classifying the partitions of trapezius for EMG localization [90]. Functionally, this definition characterizes the model muscle elements which insert on the scapular spine as middle trapezius, and those which insert on the trigonum spinae as lower trapezius. The 12 elements of serratus anterior were partitioned into equal thirds of 4 elements. The most superior partition consisted of elements connecting the 1^st^ rib with the medial scapular boarder, superior to the trigonum spinae, while the lower partition elements all originated below the 4^th^ rib, following cadaveric insights from Ekstrom et al. [87]. The middle partition elements were thus elements that did not fall under either of the classifications for the upper or lower partitions (Fig. 7). Peak joint angle differences between maximum force-generating capacity (e.g., *unweakened*) and reduced force-generating capacity (e.g., *weakened*) simulations were calculated as Δ° = *weakened* – *unweakened*, such that increases in joint angles with weakness were signed positive, and decreases in joint angles with weakness were signed negative.

## Implicit Method for Optimal Control Formulation

The implicit problem formulation described here is based on the methods described by van den Bogert, Blana, and Heinrich [53].

Multibody system dynamics can be related to system kinematics using the generalized equation of motion (Eq. 3), formulated as:

3 $$\tau =R\left({\boldsymbol{q}}\right){F}_{0}^{MT}{\boldsymbol{a}}=M\left({\boldsymbol{q}}\right)\ddot{{\boldsymbol{q}}}+C\left({\boldsymbol{q}},\dot{{\boldsymbol{q}}}\right)+G\left({\boldsymbol{q}}\right)+{J}^{T}{{\boldsymbol{\tau}}}_{end}$$

where ***τ*** is the matrix of joint forces and torques

***q***, ***q̇***, and ***q̈*** are the generalized kinematic coordinates, velocities, and accelerations, respectively

*R(****q****)* is an *n* x *m* matrix of actuator (muscle) moment arms

*F0*^*MT*^ is an *m* x *m* matrix of maximal musculotendon forces, subject to muscle length and velocity coefficients, which is multiplied by ***a***, an *m* x 1 vector of muscle activations which range from 0 (no activation) to 1 (maximum)

*M(****q****)* is the inertial matrix which is multiplied by ***q̈*** to obtain inertial force required to generate acceleration ***q̈*** and describe how the mass of a multibody system imposes joint torques which resist change in motion

*C(****q****,****q̇****)* is the vector of Coriolis and centrifugal forces that arise due to the interactions of the velocities on the individual bodies in the multibody system and the resulting joint forces

*G(****q****)* is the vector of gravity-induced joint torques resulting from the mass and posture (configuration) of the multibody system

*J*^*T*^***τ***_*end*_ is the transpose of the Jacobian* J* to map generalized joint torques *τ* to the Cartesian space approximation of the end effector which enforces consistency in dynamical systems with external forces.

To determine the state variable ***a*****,** we model it from excitation ***u*** based on the following electro-chemical model [56]:

4 $$\dot{\user2{a}} = \left( {{\boldsymbol{u}} - {\boldsymbol{a}}} \right)\left( {c_{1} {\boldsymbol{u}} + c_{2} } \right)$$

where c1 + c2 is the rate constant for activation and c2 is the rate constant for deactivation. The range of 20-50 s^-1^ has been reported depending on muscle fiber type, where in our simulations, the rate constant for across all muscles averaged at 0.47 (±0.12) s^-1^ [46, 49]. The direct collocation methods discretize the continuous state (***q***) and control (***u***) trajectories of the system dynamics into *N* nodes of equal temporal spacing along the time duration of the model motion:*for i = 1 to N-1:*

5 $${C}_{i}\equiv f\left(\frac{{{\boldsymbol{x}}}_{i+1}+ {{\boldsymbol{x}}}_{i}}{2},\frac{{{\boldsymbol{x}}}_{i+1}-{{\boldsymbol{x}}}_{i}}{{t}_{i+1}-{t}_{i}},\frac{{{\boldsymbol{u}}}_{i+1}+{{\boldsymbol{u}}}_{i}}{2}\right)=0$$

where *N* is the number of nodes for direct collocation; the system dynamics constraints at time point *i (C*_*i*_*)* are subject to approximate midpoint finite differences for: (1) the state variable vector *x*_*i*_ and *x*_*i+1*_ (first term in Eq. 5: *x*_*i+1*_* + x*_*i*_*/2*), (2) the rate of change of *x* over the interval *t*_*i*_ to *t*_*i+1*_ (second term in Eq. 5: *x*_*i+1*_*-x*_*i*_*/t*_*i+1*_* -t*_*i*_), and (3) the control variable (excitation) *u*_*i*_ and *u*_*i+1*_ (third term in Eq. 5: *u*_*i+1*_* + u*_*i*_*/2*). State and control variables are also subject to find solutions which satisfy the following constraints (Eq. 6):

6.1 $$f\left({\boldsymbol{x}},\dot{{\boldsymbol{x}}},{\boldsymbol{u}},\right)=0$$

6.2 $${u}_{Lower}\le {\boldsymbol{u}}\le {u}_{Upper}$$

6.3 $${T}_{i}\left({\boldsymbol{x}}\left(t\right),{\boldsymbol{u}}\left(t\right)\right)=0$$

where the implicit state equation *f(****x****, ****ẋ****, ****u****) = 0* enforces dynamic equilibrium across the model-predicted state variables (***q***, ***q̇***, ***s***, ***a***) and excitation (***u***), subject to constraints on muscle activity (*u*_*Lower*_*, **u*_*upper*_), and optionally constraints relative to the task such as workspace, posture, and other path constraints (*T*_***i***_). This structure determines the states, controls, and constraints that are subsequently entered into the IPOPT NLP, subject to the cost function (Eq. 1).

## Effect of Mesh Intervals on Muscle Activation Dynamics

Mesh resolution was set to 16 nodes for kinematic validation, and 8 nodes for simulating muscle weakness. This approach was chosen as processing times became very long when processing 16 nodes’ solutions with simulated muscle weakness, sometimes failing to converge within 50,000 iterations. Thus, we sought to determine the consistency of dynamic solutions between 8 node meshes by comparing the predicted activation dynamics in unweakened solutions in each plane of elevation. Since 8 node and 16 node solutions did not match temporally (Fig. 8), a piecewise linear interpolation method was used to simulate where 8 equally spaced node positions were taken from the original 16 node solution. These interpolated nodes were compared to the node values from the original 8 node optimal control solution using root-mean-squared difference (RMSD) (Table

**Table 9:** Root-mean-squared difference (RMSD) between 8 node optimal control solution and 16 node optimal control solution, linearly interpolated to 8 nodes

	Frontal Plane	Scapular Plane	Sagittal Plane
Upper Trapezius	0.128073	0.034579	0.034681
Middle Trapezius	0.086341	0.103389	0.059788
Lower Trapezius	0.140507	0.1036	0.023803
Upper Serratus Ant.	0.004333	0.069289	0.021288
Middle Serratus Ant.	0.078543	0.038081	0.027333
Lower Serratus Ant.	0.134566	0.086976	0.052913
Plane Average	**0.095394**	**0.072652**	**0.036634**

^9^). The largest RMSD values were calculated for frontal plane simulations, specifically upper trapezius, lower trapezius, and lower serratus anterior activation dynamics. Average RMSD across all planes and trials was 0.068.

We expect that mesh density, while coarse, retained dynamic consistency due to the slow and simple movements that we prescribed. Thus, states were not expected to change drastically between nodes. All simulations, whether 8 or 16 nodes, were set to achieve an optimality tolerance and feasibility tolerance of 1.00e−3.

## Scapulothoracic Contact Forces

Contact between the scapula and the thorax segments was modeled as an elastic force. The contact surface of the thorax was modeled as an ellipsoid, with two contact points on the scapula located at the (1) trigonum spinae and the (2) angulus inferior. Contact forces rose rapidly if these points penetrated the thorax and were attenuated if these points egressed beyond the perimeter of the thorax. The result is high contact forces during penetration and low contact forces during egress. The full mathematical formulation and derivation of these forces are published previously [50]. In our simulations, these forces were always less than 1N; thus, comparisons between planes and ‘weakness’ conditions are minimal (Fig. 9A-C).
